# Artificial Intelligence and Novel Technologies for the Diagnosis of Upper Tract Urothelial Carcinoma

**DOI:** 10.3390/medicina61050923

**Published:** 2025-05-20

**Authors:** Nikolaos Kostakopoulos, Vasileios Argyropoulos, Themistoklis Bellos, Stamatios Katsimperis, Athanasios Kostakopoulos

**Affiliations:** 1 1st Department of Urology, Metropolitan General Athens, 15562 Athens, Greece; nikostakop@gmail.com (N.K.); argvas@otenet.gr (V.A.); 2 2nd Department of Urology, University of Athens, 15126 Athens, Greece; bellos.themistoklis@gmail.com (T.B.); stamk1992@gmail.com (S.K.)

**Keywords:** urothelial carcinoma, artificial intelligence, upper tract urothelial carcinoma, urothelial carcinoma diagnosis

## Abstract

*Background and Objectives*: Upper tract urothelial carcinoma (UTUC) is one of the most underdiagnosed but, at the same time, one of the most lethal cancers. In this review article, we investigated the application of artificial intelligence and novel technologies in the prompt identification of high-grade UTUC to prevent metastases and facilitate timely treatment. *Materials and Methods*: We conducted an extensive search of the literature from the Pubmed, Google scholar and Cochrane library databases for studies investigating the application of artificial intelligence for the diagnosis of UTUC, according to the PRISMA guidelines. After the exclusion of non-associated and non-English studies, we included 12 articles in our review. *Results*: Artificial intelligence systems have been shown to enhance post-radical nephroureterectomy urine cytology reporting, in order to facilitate the early diagnosis of bladder recurrence, as well as improve diagnostic accuracy in atypical cells, by being trained on annotated cytology images. In addition to this, by extracting textural radiomics features from data from computed tomography urograms, we can develop machine learning models to predict UTUC tumour grade and stage in small-size and especially high-grade tumours. Random forest models have been shown to have the best performance in predicting high-grade UTUC, while hydronephrosis is the most significant independent factor for high-grade tumours. ChatGPT, although not mature enough to provide information on diagnosis and treatment, can assist in improving patients’ understanding of the disease’s epidemiology and risk factors. Computer vision models, in real time, can augment visualisation during endoscopic ureteral tumour diagnosis and ablation. A deep learning workflow can also be applied in histopathological slides to predict UTUC protein-based subtypes. *Conclusions*: Artificial intelligence has been shown to greatly facilitate the timely diagnosis of high-grade UTUC by improving the diagnostic accuracy of urine cytology, CT Urograms and ureteroscopy visualisation. Deep learning systems can become a useful and easily accessible tool in physicians’ armamentarium to deal with diagnostic uncertainties in urothelial cancer.

## 1. Introduction

Upper tract urothelial cancer (UTUC) is one of the most aggressive but under-studied tumours in both sexes and includes neoplasms of the ureters and the pelvicalyceal system. It is associated with very high mortality in advanced stages, and thus, there is an increased need to improve imaging modalities which will facilitate early diagnosis [1].

Nowadays, initial diagnoses of UTUC and upper urinary tract recurrence after conservative or radical management are achieved with urine cytology reporting, computed tomography urogram (CTU) findings and semirigid and flexible diagnostic ureteroscopy. Nevertheless, none of these techniques have managed to provide high diagnostic accuracy, especially in identifying flat lesions, which, for example, are generally not visible with CT, while CT Urograms also lack accuracy in diagnosing the pathological grade of tumours [1].

In addition to this, ureteroscopic biopsies, although able to determine tumour grade in over 90% of cases, are responsible for significant undergrading and understaging, which causes inaccurate risk stratification, when compared to RNU specimens. On top of this, flexible ureteroscopy can be challenging to perform, especially when searching for tumours inside the pelvicalyceal system [1].

During the last few years, there have been significant advancements in the application of artificial intelligence (AI) in the medical field, and especially in improving the diagnosis of aggressive cancers, such as ovarian, prostate and bladder cancer [2,3,4,5,6]. Artificial intelligence is defined as the capability of computational systems to perform tasks that are traditionally expected to be performed by humans [2].

However, studies demonstrating the effective use of AI technology to improve the diagnostic accuracy of available imaging modalities in order to promptly identify upper tract urothelial carcinoma are still lacking. In this review article, we research the available literature, and we present, for the first time to the best of our knowledge, a narrative review about the novel technologies available in urologists’ armamentarium, which can provide timely diagnoses of upper urothelial tract carcinoma.

Furthermore, we investigate and discuss the potential role of artificial intelligence in facilitating the selective and effective identification of aggressive disease, in order to decrease the delayed diagnosis of metastatic UTUC as well as overtreatment.

## 2. Materials and Methods

**Figure 1:**
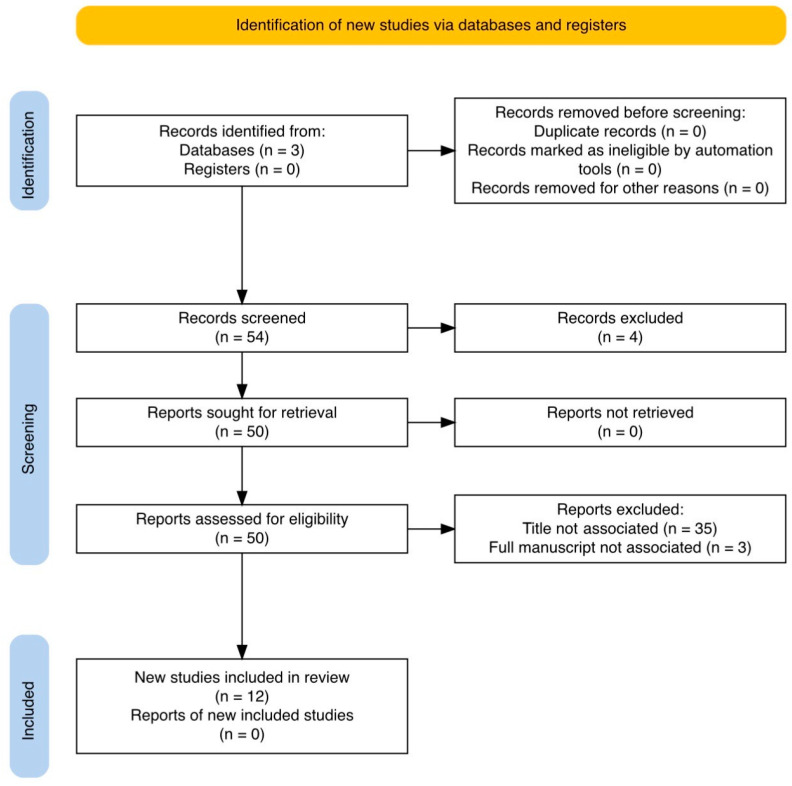
PRISMA guidelines flowchart.

Extensive research was conducted including all available studies from the Pubmed, Google scholar and Cochrane library databases, using the associated keywords “upper tract urothelial carcinoma” AND “artificial intelligence” AND “diagnosis” OR “diagnosis of upper tract urothelial carcinoma”, without restrictions concerning the date of publication, according to the PRISMA guidelines (Figure 1).

The inclusion criteria for articles were that they involved artificial intelligence application for the diagnosis of UTUC or the diagnosis of recurrence after conservative or radical treatment of UTUC. Our initial search retrieved 54 studies. After the initial title and abstract screening, we excluded duplicates, non-English studies and articles not associated with our criteria.

Studies involving non-AI methods such as statistical analysis methods and existing nomograms were excluded from the full manuscript screening process. In addition to this, studies focusing on other cancers or bladder cancer as the initial diagnosis, or generally focusing on urothelial cancer without clarifying the inclusion of UTUC cases, were also excluded, as were studies not concerning exclusively the diagnosis of UTUC. After the final full manuscript screening, 12 studies were selected to be included in our review article.

Title and abstract screening was conducted by two independent reviewers (NK and VA), and disagreements were resolved by a third reviewer (AK). The selected studies were assessed by three different reviewers (NK, TB, SK), and the Results and Discussion sections were written by them. The final reviewing and correction of the manuscript were independently performed by two reviewers (VA, AK).

## 3. Results

After the screening process, 12 studies were included in our review article [Table 1]. Eleven of the selected studies were retrospective in design, with a relatively small number of patients (6–483 patients), with a mean of 13,673 patients with UTUC [7,8,9,10,11,12,13,14,15,16,17]. Only one selected study was a review article, which presented the technologies available to improve the performance of urine cytology [18]. Of the selected studies, two retrospective studies and one review article were associated with urine cytology [7,8,18]; three retrospective studies presented deep learning models for computed urogram findings [9,10,11]; two retrospective studies were about diagnostic ureteroscopy [12,13]; and four retrospective studies concerned ChatGPT artificial intelligence [14], histopathological slide interpretation [15], inflammatory marker pre-radical nephroureterectomy (RNU) [16] and *N*-glycans scoring systems [17].

The available literature presents promising retrospective data concerning artificial intelligence system application for the successful and timely diagnosis of UTUC. The most crucial applications have been shown to be associated with post-radical nephroureterectomy urine cytology reporting [7,8] and with deep learning systems that can be trained to improve ureteroscopic vision [12,13], as well as extract textural radiomics features from computed tomography urograms, to predict UTUC tumour grade and stage in <2 cm tumours [9,10,11].

Artificial intelligence, such as ChatGPT, has been proven helpful in providing patients with general information about the risk factors and epidemiology of UTUC [14], while deep learning workflow applied in histopathological slides can predict UTUC protein-based subtypes [15].

The use of machine learning has been shown to yield promising results from a novel pre-RNU systemic immune-inflammation score (SIIS) nomogram, as an independent predictor of prognosis in patients with UTUC [16], as well as in creating urological disease-specific scoring systems with the use of biomarkers such as*N*-glycans [17].

## 4. Discussion

Although UTUC is a relatively rare type of cancer, accounting for only 5–10% of urothelial cancers, it is commonly diagnosed in advanced stages, with 60% being aggressive upon diagnosis and 30% metastatic [1,9]. Intravesical recurrence after radical nephroureterectomy (RNU) for the treatment of UTUC is very high, with rates of 22–47% [1]. Thus, strict follow-up with cystoscopy and urine cytology is necessary. Using artificial intelligence to increase urine cytology sensitivity has been suggested recently [7]. The AIxURO system is a deep learning model that performs segmentation and is aligned with the guidelines in the Paris System 2.0 for urine cytology, particularly in order to distinguish between high-risk “suspicious cells” and low-risk “atypical cells” in urine cytology samples of patients that have undergone RNU.

The AIxURO system increased postoperative urine cytology reporting confidence and managed to diagnose intravesical recurrence in misdiagnosed patients from their urine cytology samples [7]. In a retrospective study by Chen CC. et al. [7], the results of postoperative digitised cytology slides, assessed by the AIxURO system, were compared to cytopathologists’ reports.

With the assistance of the artificial intelligence system AIxURO, bladder tumour recurrence post RNU was detected in 37% (10/27) of patients by combining advanced urine analysis with artificial intelligence techniques, whereas traditional urine cytology examination identified recurrences in only 29.6% (8/27) of patients. Hence, AIxURO showed greater sensitivity and accuracy in comparison to traditional urine cytology, although the authors admit that their study had many limitations, such as the small sample size and retrospective study design [7].

These findings were confirmed by a larger study of post-RNU cytologies evaluated by AIxURO [8]. The AIxURO system provided more accurate and consistent results, especially in atypical cell samples, in comparison to the assessments by a cytopathologist and a cytotechnologist. The cooperation of artificial intelligence with experienced clinicians’ has been proposed to reduce diagnostic discrepancies and significantly improve the reliability of the urine cytology diagnosis of UTUC bladder recurrence after RNU [8].

A significant correlation has been proven between the pathological grade of UTUC and prognosis of the disease, with a higher grade associated with a poorer prognosis. Hence, precise preoperative identification of the UTUC pathological stage could significantly improve the survival of patients [9].

Radiomics technology, by using a random forest machine learning (MC) algorithm based on a computed tomography urogram (CTU), could greatly assist in the prediction of high-grade UTUC and potentially reduce unnecessary invasive procedures to some extent.

Many studies have proposed the construction of nomogram models to predict high-grade UTUC by extracting images from CTU [9,10,11]. In one study, the maximal tumour diameter was recorded in three phases from CTU images (unenhanced, medullary, excretory) by using the Radiomics module of the 3D Slicer software (version 5.0.3) [9].

The segmentation was performed manually for each tumour by a specialised radiologist. The radiomics scores obtained from the mixed-phase characteristics of 167 patients showed statistically significant differences between low-grade and high-grade UTUC (*p* < 0.05). The nomogram showed a probability of predicting high-grade UTUC of 96.8% and compared to urine cytology, it achieved higher accuracy and sensitivity. However, it also showed lower specificity, which limits nomograms’ ability to replace postoperative tissue pathological examination [9].

In another study by Alqahtani A. et al. [10], using the same radiomics technology, the authors managed to successfully differentiate high-grade from low-grade tumours, as well as early-stage from advanced UTUC. The application of machine learning and enhanced CT Urograms is suggested as the beginning of “virtual biopsy”, which the authors showed performed significantly better than ureteroscopic histopathology in small (<2 cm) UTUC stage and grade prediction. Future prospective studies with larger populations are certainly needed to standardise these radiomics-based models [10].

The random forest (RF) machine learning model showed the best performance in predicting high-grade UTUC, in comparison to other machine learning models, upon extracting features from three periods of CTU images. It also had the highest area-under-the-curve (AUC) values, with 0.914 (95% Confidence Interval [95% CI] = 0.852–0.977) and 0.903 (95% CI = 0.809–0.997), in the training set and validation set, and accuracies of 0.878 and 0.857, respectively. Hydronephrosis has been proven the best independent influencing factor for high-grade disease [11].

In addition to this, a well-recognised difficulty during the conservative treatment of UTUC via ureteroscopic laser ablation is poor endoscopic visibility, due to bleeding from the tumour and a narrow ureteral lumen [12]. U-net computer vision models have been proven to augment visualisation during treatment. By comparing 20 different videos of patients who received endoscopic treatment, the U-net system managed to successfully segment both the UTUC tumours and the areas of ablation. These deep learning computer vision models have demonstrated excellent real-time performance for automated UTUC segmentation during ureteroscopy, facilitating more accurate conservative treatment [12].

Computer-assisted interventions (CAIs) can potentially provide information to urologists during diagnostic ureteroscopies for UTUC, or during the treatment of UTUC with ureteroscopic laser ablation. Several navigation systems have been proposed and could be considered precursors to robotic ureteroscopy. CAI has been shown to provide spatial–temporal information to improve hollow lumen segmentation in ureteroscopic images, especially in challenging situations such as cases with poor visibility, occasional bleeding and specular reflections [13].

Large language models (LLM) such as ChatGPT, which are powered by artificial intelligence, have been suggested to potentially assist in the medical diagnosis of several diseases in the near future [14].

However, their use is still limited because of the reproducibility of author bias and potential spread of misinformation.

A study evaluated the responses of ChatGPT in regard to patients’ questions associated with UTUC, such as the risk factors of the disease, as well as its definition, most common symptoms and treatment methods [14].

It was shown that ChatGPT had suboptimal performance, especially in the questions associated with the treatment of UTUC. It achieved its highest scores concerning the risk factors of UTUC.

Thus, ChatGPT can be considered a very useful tool in assisting the public in understanding basic aspects of the disease and gain general knowledge of prophylaxis against UTUC. It is certain that ChatGPT cannot replace proper medical care and assessment by urologists, but nevertheless, it can simplify information concerning the disease for patients [14].

The use of artificial intelligence has also been proposed to predict UTUC protein-based subtypes, by developing a deep learning workflow (DL) in order to identify them directly from histopathological H&E slides [15].

In a retrospective study by Angeloni M. et al. [8], these subtypes were identified using the immunohistochemical expression of three luminal (FOXA1, GATA3 and CK20) and three basal (CD44, CK5 and CK14) markers with the assistance of a DL model based on a transfer learning approach by fine-tuning a pre-trained ResNet50. The DL model successfully predicted protein- based UTUC subtypes, with basal predictions mainly containing PD-L1-positive samples, and luminal predictions containing a higher proportion of FGFR-3-mutated samples [15].

Other novel technologies involving artificial intelligence have also shown promising results in recent years [16]. A novel pre-RNU systemic immune-inflammation score (SIIS) nomogram, with the assistance of machine learning, has been found to be an independent predictor of prognosis in patients with UTUC [16].

An SIIS-related nomogram was created to predict 1-, 3- and 5-year overall survival in UTUC patients using a Cox regression model and machine learning with a random survival forest model. A higher SIIS-score which consists of a combination of a higher preoperative serum neutrophil-to-lymphocyte ratio, monocyte-to-lymphocyte ratio, platelet-to-lymphocyte ratio, systemic immune-inflammation index and systemic inflammation response index was associated with significantly shorter overall survival [16].

Machine learning urological disease-specific scoring systems that use biomarkers such as *N*-glycans, specifically monogalactosyl biantennary *N*-glycan (G1) and agalactosyl bisecting GlcNAc core fucosyl *N*-glycan (G0FB), have been shown to have a high impact on the early detection of UTUC [17].

Furthermore, these biomarkers are able to discriminate between bladder cancer and UTUC, in order to select a disease-specific treatment, using only one-time serum collection [17].

To summarise the available data from the literature, it is well established that the diagnosis of UTUC is challenging, even in recent years, because of the limited accuracy of the available diagnostic tools [18].

The performance of the Paris system for Reporting Urine Cytology (TPS) in UTUC is hindered by the difficult-to-access location of tumours, instrumentation artefacts and ureteroscopic trauma. This results in poor overall performance, especially for low-grade UTUC, while for high-grade disease, urine cytology demonstrates unreliable sensitivity and high specificity. Based on these findings, artificial intelligence and machine learning may facilitate the objective application of TPS criteria to urine samples for early UTUC diagnosis, as well as for its distinction from bladder cancer [18].

This is the first literature review, to the best of our knowledge, to summarise all of the recent updates in the application of artificial intelligence technologies and machine learning models for the successful diagnosis of UTUC. Other reviews have already addressed the application of AI for urothelial carcinoma in general [20] or for other aspects of UTUC, such as the prediction of the most crucial prognostic factors of the disease [21].

The available studies were mostly retrospective in design and generally had a small number of patients taking part, which is the main limitation of our review article. This is also a comprehensive rather than systematic review; in the future, the latter could be conducted to improve the level of available evidence on the use of AI. Moreover, although ethical approval was obtained in all the included studies, it is important that patients are closely consulted about the risks of fully trusting diagnostic findings which are based on AI, since it is a field of technology that is still evolving and requires improvements.

## 5. Conclusions

The application of artificial intelligence and machine learning models has been shown to be successful in facilitating the timely identification of high-grade UTUC by improving the diagnostic accuracy of urine cytology, CT Urograms and ureteroscopy visualisation. Most of these novel artificial intelligence technologies have not yet been validated for routine clinical practice, and should first be evaluated in larger groups of patients. However, the initial clinical experience from their use indicates that they could become a useful and easily accessible tool in urologists’ everyday clinical practice in the very near future to deal with upper tract urothelial cancer diagnostic uncertainties.

## 6. Future Directions

Currently, none of the AI technologies described in this review article have been validated for routine clinical use, nor have they been included in clinical guidelines. Larger comparative studies of the AIxURO deep learning system versus traditional urine cytology are very likely to prove its value in everyday UTUC diagnostic algorithms, since it has already demonstrated greater sensitivity and accuracy in comparison to traditional urine cytology [7,8].

In the same way, radiomics machine learning models are very close to being incorporated into the current imaging modality protocols [9,10,11], whereas other artificial intelligence applications, such as technologies to enhance ureteroscopic visualisation and diagnostic biomarkers, are still in the early experimental stage [12,13,15,16,17].

ChatGPT, although authorised to be used by patients, is not yet considered reliable by the medical community, so patients should not trust it for clinical judgement but only for general epidemiological data and for suggestions on preventive methods [14].

Randomised prospective studies with larger populations are needed to verify the above findings and standardise the use of these novel technologies in clinical practice guidelines, in the same way as has already been initiated, in general, for urothelial carcinoma with the prospective validation of study results concerning the use of artificial intelligence [22].

## Figures and Tables

**Table 1:** Artificial intelligence applications for the diagnosis of UTUC. * Radiomics: A technology that extracts and analyses quantitative data from medical images using machine learning algorithms [9,10]. ** Machine learning: A type of artificial intelligence that develops statistical algorithms in order to generalise data information to unseen data and perform tasks without the need for instructions [9,10,11,19]. *** Deep learning: A subcategory of machine learning that uses multiple layers in a network and is specialised in performing classification and representation learning [19].

Study	Number of Patients (Mean: 13,673)	AI Application Type for UTUC Diagnosis	Type of Study
Chen CC et al. [7]	113	Urine cytology diagnostic accuracy	Retrospective study
2. Chang KY et al. [8]	185	Urine cytology diagnostic accuracy	Retrospective study
3. Zheng Y et al. [9]	140	Radiomics * CTU nomogram	Retrospective study
4. Alqahtani A et al. [10]	106	Radiomics * CTU nomogram	Retrospective study
5. Zheng Y et al. [11]	167	Machine learning ** CTU model	Retrospective study
6. Lu D et al. [12]	20	Ureteroscopic vision enhancement	Retrospective study
7. Lazo JF et al. [13]	6	Ureteroscopic vision enhancement	Retrospective study
8. Łaszkiewicz J et al. [14]	16	ChatGPT performance	Retrospective study
9. Angeloni M et al. [15]	163	Histopathology slide deep learning system ***	Retrospective study
10. Liu J et al. [16]	483	Systemic immune-inflammation score machine learning **	Retrospective study
11. Iwamura H et al. [17]	105	Immunoglobulin N-glycan machine learning **	Retrospective study
12. Chukwudebe O et al. [18]	n/a	Urine cytology diagnostic accuracy	Narrative review
